# Characterisation, electrochemical and oxidative addition data of organophosphorus-containing rhodium(I) complexes

**DOI:** 10.1016/j.dib.2018.10.103

**Published:** 2018-10-27

**Authors:** P.S. Manana, E. Erasmus

**Affiliations:** Department of Chemistry, University of the Free State, Bloemfontein 9300, South Africa

## Abstract

This data article contains the ^1^H Nuclear Magnetic Resonance (NMR), ultraviolet and visible (UV–vis) and Attenuated Total Reflectance Fourier Transformed Infra-red (ATR FTIR) characterization of a series of organophosphorus-containing rhodium(I) complexes. The electrochemical data acquired by means of cyclic voltammetry of the three organophosphorus-containing ligands (with the structure C_6_H_5_XPPh_2_, where X = O, S and NH) and their acetylacetonato (monocarbonyl) organophosphorus rhodium(I) compounds, [Rh(acac)CO(C_6_H_5_XPPh_2_)] are reported. Additionally, the kinetic data of the oxidative addition of methyl iodide to the rhodium(I) complexes, are also presented.

**Specifications table**

**Table:** 

Subject area	*Chemistry*
More specific subject area	*Metal complexes, Cyclic voltammetry, kinetics*
Type of data	*Table, figure*
How data were acquired	*NMR were recorded on a Bruker Avance DPX 300 NMR spectrometer, ATR FTIR were recorded on a Nicolet IS50 FTIR Tri-detector gold spectrometer, with a build-in diamond ATR module as well as in a KCl liquid cell connected to a water bath for temperature control, Cyclic voltammograms were recorded on a Princeton Applied Research PARSTAT 2273 voltammograph, running PowerSuite (Version 2.58), UV–vis were recorded on a Shimadzu UV/Vis spectrometer*
Data format	*Analyzed*
Experimental factors	*All electrochemical data are reported, using the potential of the ferrocene/ferricinium redox couple [FcH/FcH*^*+*^*] (E*^*°`*^*= 0.00 V) as an internal reference.*
	*The kinetic measurements were recorded at four temperatures ranges between 15 and 45 °C.*
Experimental features	Electrochemical measurements were conducted on *ca.* 0.2 mmol dm^-3^ solutions of the analyte in acetonitrile, containing 0.10 mmol dm^-3^ tetra-*n-*butylammonium hexafluorophosphate as supporting electrolyte.
	All kinetic measurements were monitored under pseudo first-order conditions with a 500–2000 times molar excess of CH_3_I over the concentration of the rhodium complex
Data source location	*Department of Chemistry, University of the Free State, Bloemfontein, 9300, Republic of South Africa*
Data accessibility	*Data are presented in this article*
Related research article	*Erasmus E, Synthesis and unexpected electrochemical reaction of p-substituted phenyl diphenylphosphinites, J. Electroanal. Chem. 2014 727:1-7*
	*Cheung W-M, Lai C-T, Zhang Q-F, Wong W-Y, Williams ID, Leung W-H, Iridium and rhodium complexes containing dichalcogenoimidodiphosphinato ligands, Inorg. Chim. Acta, 2006 359:2712-2720*

**Value of the data**

• The data can be used for comparison with related compounds; here, it is demonstrated that when changing the X in the ligand structure C_6_H_5_XPPh_2_ with O, S and NH, the electrochemical response of both the ligand and the Rh(I) complex.
• The data can be used during catalyst design to compare how variation of X in the ligand structure C_6_H_5_XPPh_2_ with O, S and NH, influences the oxidative addition of methyl iodide to the rhodium(I) complexes.
• The data can be used for comparison with related compounds; here, we present the ^1^H NMR, ^31^P NMR, UV–vis and ATR FTIR spectroscopy which provides characterisation data for research community on organophosphorus ligands and their Rh(I) complexes.

## 1 Data

**Fig. 1:**
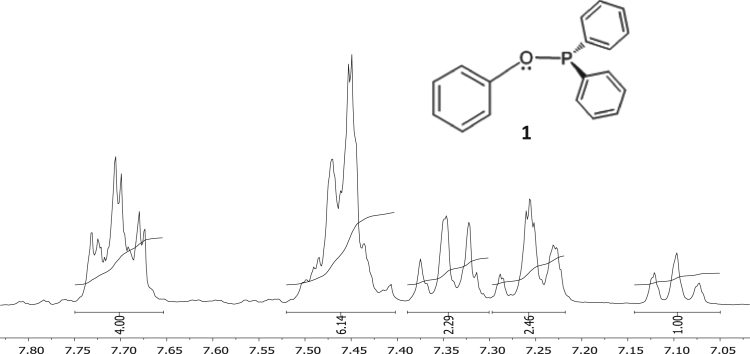
^1^H NMR of phenyl diphenylphosphinite, C_6_H_5_OPPh_2_, **1**.

**Fig. 2:**
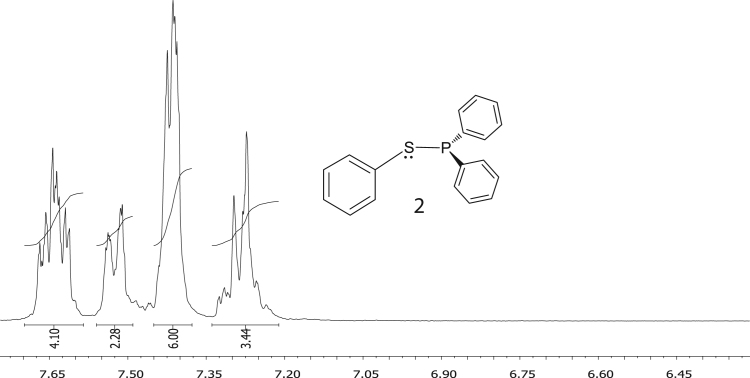
^1^H NMR of diphenylphosphinothious acid, C_6_H_5_SPPh_2_, **2**.

**Fig. 3:**
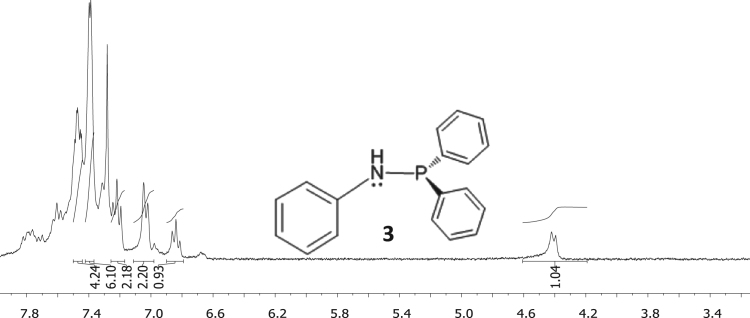
^1^H NMR of diphenylphosphino amide C_6_H_5_NHPPh_2_, **3**.

**Fig. 4:**
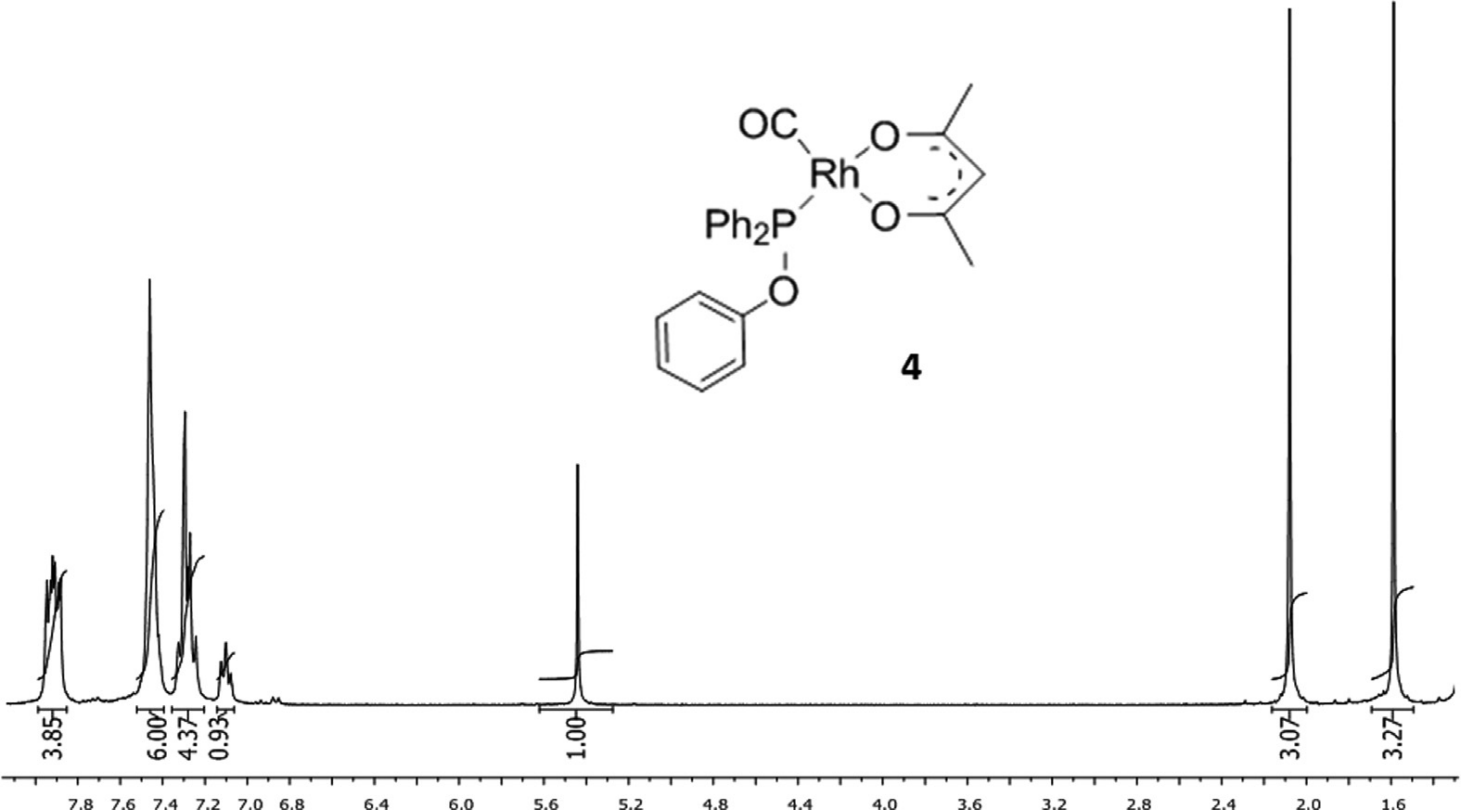
^1^H NMR of [Rh(acac)CO(C_6_H_4_OPPh_2_)]*,***4**.

**Fig. 5:**
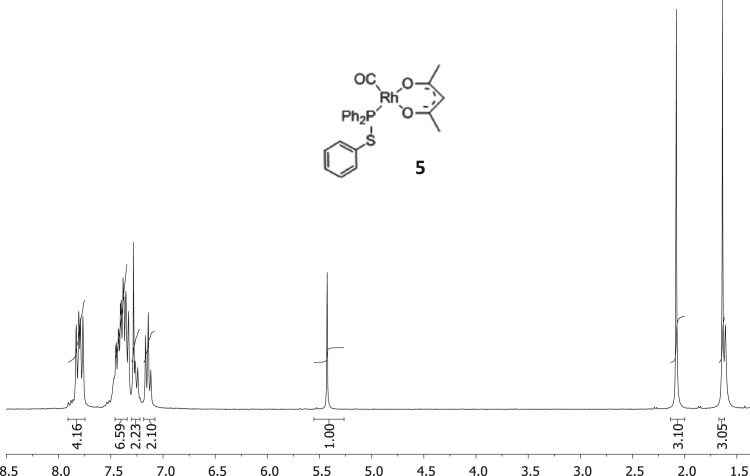
^1^H NMR of [Rh(acac)CO(C_6_H_4_SPPh_2_)], **5**.

**Fig. 6:**
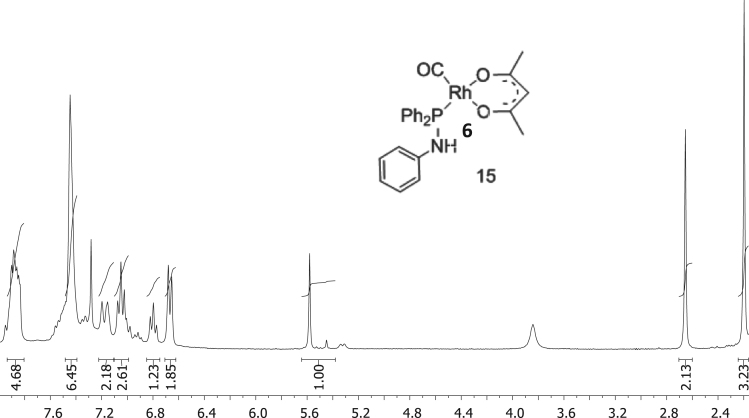
^1^H NMR of [Rh(acac)CO(C_6_H_4_NHPPh_2_)], **5**.

**Fig. 7:**
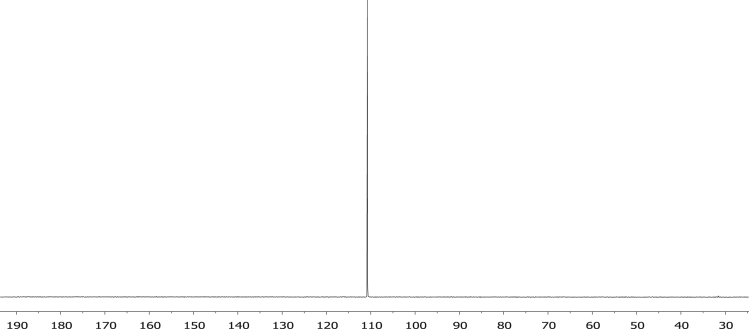
^31^P NMR of phenyl diphenylphosphinite, C_6_H_5_OPPh_2_, **1**.

**Fig. 8:**
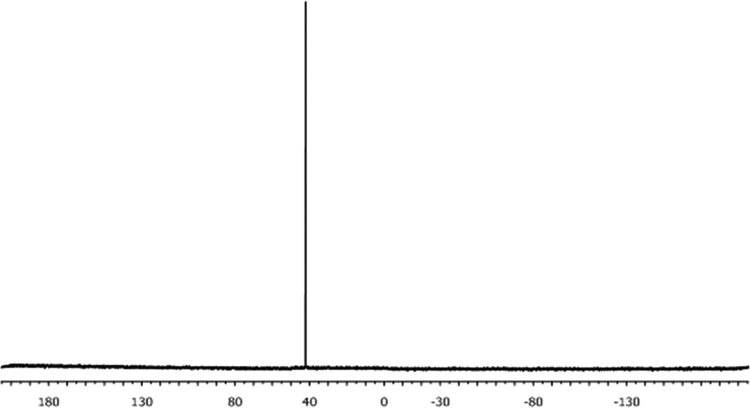
^31^P NMR of diphenylphosphinothious acid, C_6_H_5_SPPh_2_, **2**.

**Fig. 9:**
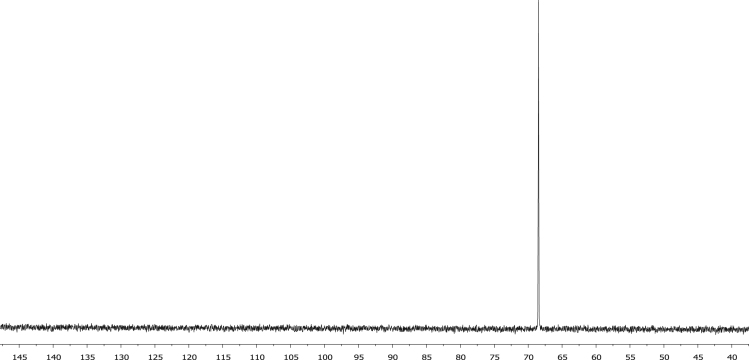
^31^P NMR of diphenylphosphino amide C_6_H_5_NHPPh_2_, **3.**

**Fig. 10:**
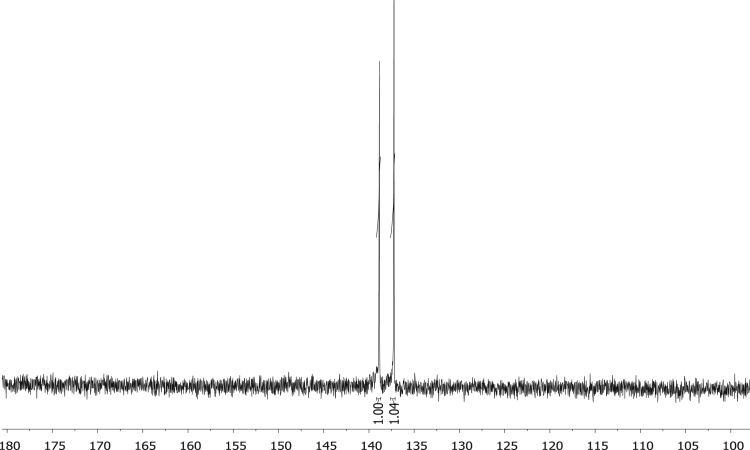
^31^P NMR of [Rh(acac)CO(C_6_H_4_OPPh_2_)], **4**.

**Fig. 11:**
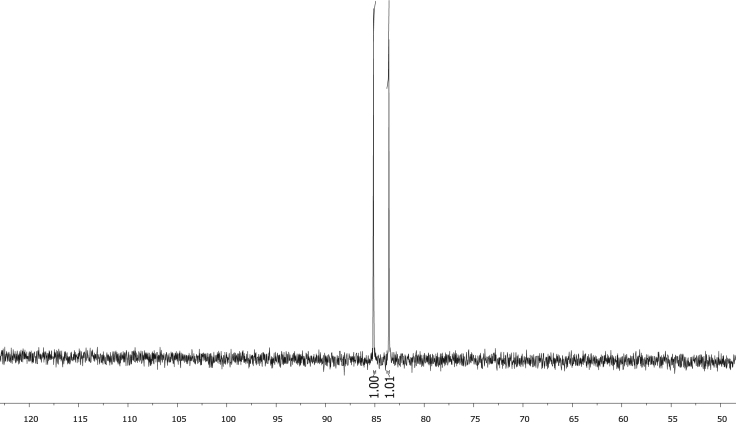
^31^P NMR of [Rh(acac)CO(C_6_H_4_SPPh_2_)], **5**.

**Fig. 12:**
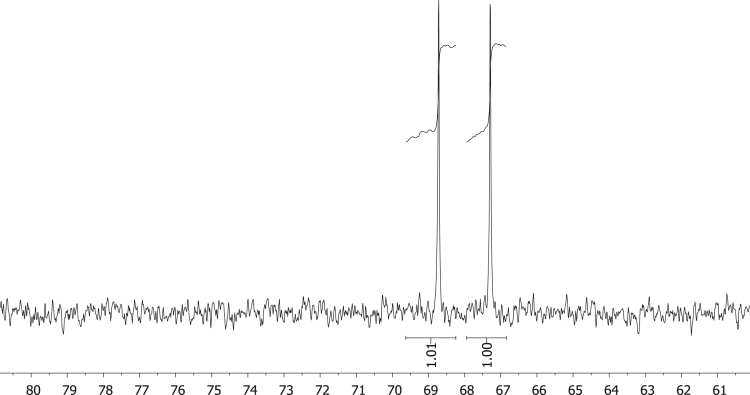
^31^P NMR of [Rh(acac)CO(C_6_H_4_NHPPh_2_)], **5**.

**Fig. 13:**
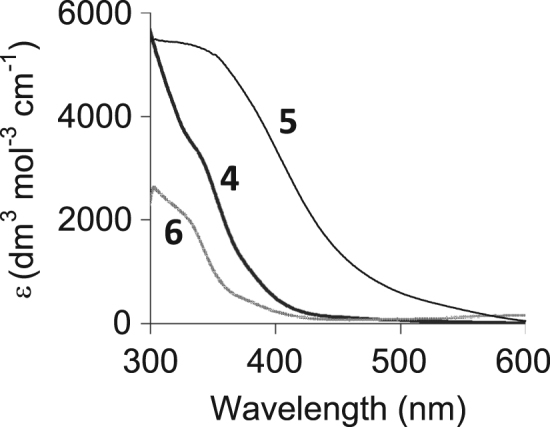
UV/vis spectra of the rhodium(I) complexes Rh(H_3_CCOCHCOCH_3_)CO(C_6_H_5_XPPh_2_), where X = O (**4**), S (**5**) and NH (**6**) at 25 °C in chloroform.

**Fig. 14:**
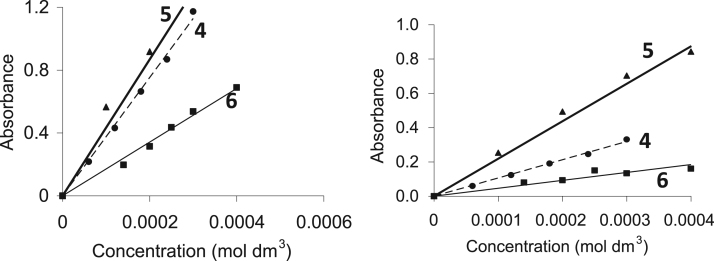
Graph of absorbance vs concentration of **4**, **5** and **6** at 25 °C in chloroform taken at λ_exp_ = 330 nm (left) and λ_exp_ = 380 nm (right) as indicated use to validate the Beer–Lambert law.

The characterisation by means of Nuclear Magnetic Resonance (^1^H NMR see Fig. 1, Fig. 2, Fig. 3, Fig. 4, Fig. 5, Fig. 6 and ^31^P NMR see Fig. 7, Fig. 8, Fig. 9, Fig. 10, Fig. 11, Fig. 12), ultraviolet and visible (see Fig. 13 and Table 1 for the UV–vis spectra, the Beer–Lambert law is shown in Fig. 14) and Attenuated Total Reflectance Fourier Transformed Infra-red (see Table 1, for the summary of the ATR FTIR data) of three organophosphorus ligands with the structure C_6_H_5_XPPh_2_, where, X = O (**1**), S (**2**) and NH (**3**) and their acetylacetonato (monocarbonyl) organophosphorus rhodium(I) compounds, [Rh(acac)CO(C_6_H_5_XPPh_2_)] where X = O (**4**), S (**5**) and NH (**6**) are presented.

**Table 1:** Carbonyl stretching frequencies and molecular extinction coefficients (ε) of the rhodium(I) complexes [Rh(H_3_CCOCHCOCH_3_)CO(C_6_H_5_XPPh_2_) where X = O (**4**), S (**5**) and NH (**6**) at 25 °C in chloroform (λ_exp_ = λ_maks_).

	*No.*	ʋ(CO) cm^−1^	λ_exp_/nm	ε/dm^3^ mol^−1^ cm^−1^
Rh(H_3_CCOCHCOCH_3_)CO(C_6_H_5_OPPh_2_)	***4***	1982	330	3591
Rh(H_3_CCOCHCOCH_3_)CO(C_6_H_5_SPPh_2_)	***5***	1979	330	5390
Rh(H_3_CCOCHCOCH_3_)CO(C_6_H_5_NHPPh_2_)	***6***	1981	330	2202

**Fig. 15:**
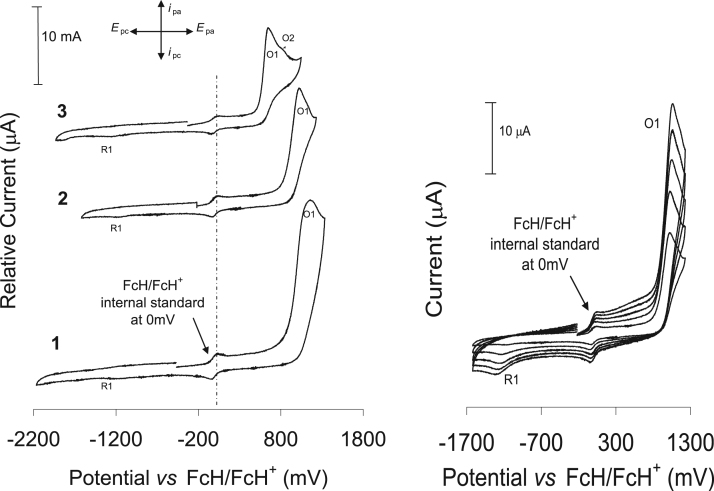
Left: a comparative graph of the cyclic voltammograms of 0.2 mmol dm^−3^ of the organophosphorus ligands (**1–3**) in CH_3_CN/0.1 mol dm^−3^ [NBu_4_][PF_6_], on a glassy carbon working-electrode, at 25 °C, and a scan rate of 100 mV s^−1^. Right: cyclic voltammogram of **2**, in acetonitrile on a glassy carbon working electrode at 25 °C and at scan rates of 100–500 mV s^−1^ (100 mV increments).

**Fig. 16:**
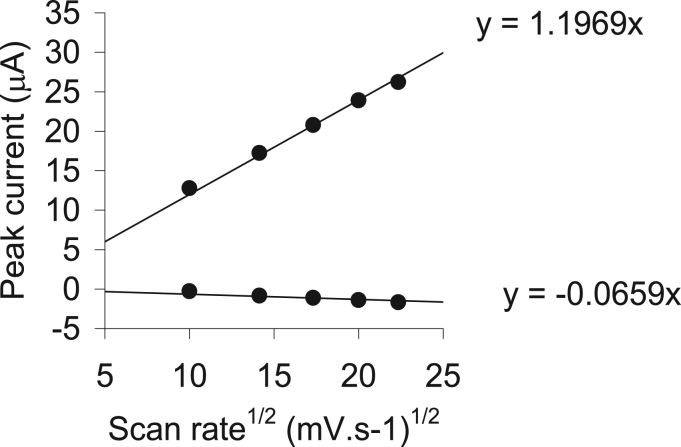
Graph illustrating the linear relationship between the anodic and cathodic peak currents and (scan rate)^1/2^ for ligand **2** as an example.

**Fig. 17:**
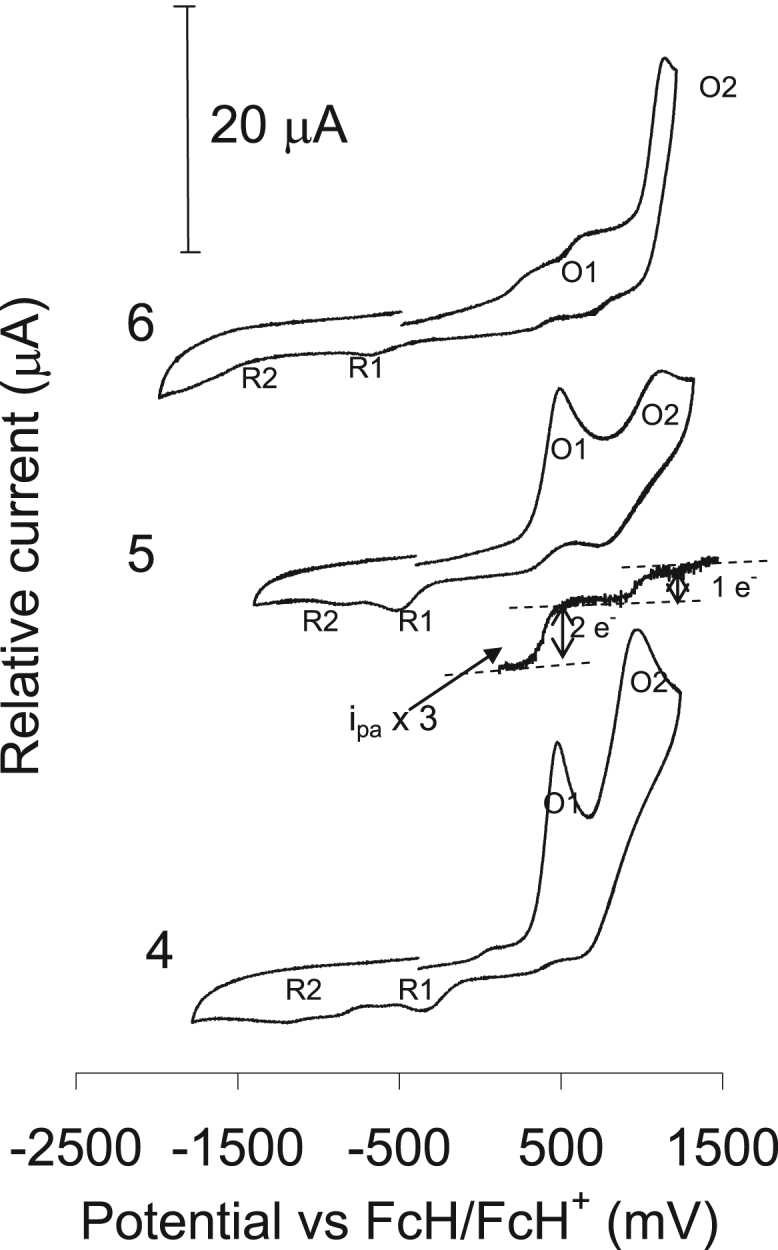
A comparative graph of the cyclic voltammograms of 0.2 mmol dm^−3^ of the rhodium (I) complexes (**4–6**) in CH_3_CN/0.1 mol dm^−3^ [NBu_4_][PF_6_], on a glassy carbon working-electrode, at 25 °C, and a scan rate of 100 mV s^−1^. The linear square wave of **4** is also shown just above the CV of **4**.

The electrochemical behavior is presented as comparative graphs of the cyclic voltammograms of ligands **1–3** (see Fig. 15 and Fig. 16 with the data reported in Table 2), while the acetylacetonato (monocarbonyl) organophosphorus rhodium(I) compounds, **4–6**, are given in Fig. 16 and the data are summarized in Table 3 (Fig. 17).

**Table 2:** The data obtained for a 0.2 mM solution of the organophosphorus ligands (**1–3**) in CH_3_CN/0.1 mol dm^−3^ [NBu_4_][PF_6_] at 25 °C, at different scan rates and reference against FcH/FcH^+^ as the internal standard. The diffusion coefficient, D, E_pa_ (anodic peak potential) as well as i_pa_ (anodic peak current and, E_pc_ (cathodic peak potential) peak for each compound is shown.

Name	No.	D for i_pa_ and i_pc_ (cm^2^ s^−1^)	ʋ/mV s^−1^	E_pa_/mV	i_pa_/µA	E_pc_/mV
C_6_H_5_OPPh_2_	**1**	6.07 × 10^-−5^	100	1166	16.9	−1257
			200	1179	22.0	−1570
			300	1192	24.1	−1737
		2.8 × 10^−11^				
			400	1205	24.6	−1765
			500	1216	26.2	−1778
C_6_H_5_SPPh_2_	**2**	5.01 × 10^−5^	100	1030	12.2	−1199
			200	1043	17.2	−1237
			300	1056	20.8	−1275
		1.52 × 10^−7^				
			400	1069	23.9	−1313
			500	1083	26.2	−1350
C_6_H_5_NHPPh_2_	**3**	3.24 × 10^−5^	100	652	10.4	−1272
			200	657	14.6	−1304
			300	662	17.9	−1307
		1.3 × 10^−11^				
			400	667	21.0	−1315
			500	674	23.2	−1323

**Table 3:** The data obtained for a 0.2 mM solution of the rhodium complexes (**4–6**) in CH_3_CN/0.1 mol dm^−3^ [NBu_4_][PF_6_] at 25 °C, reference against FcH/FcH^+^ as the internal standard at a scan rate of 200 mV s^−1^.

No.	E_pa_ (O1)/mV	i_pa_/µA	E_pa_ (O2)/mV	i_pa_/µA	E_pc_ (R1)/mV	E_pc_ (R2)/mV
**4**	461	11.8	1034	5.1	−431	−1224
**5**	527	13.2	1148	9.4	−555	−891
**6**	375 (676)	1.2 (1.1)	1179	10.1	−676	−1318

**Fig. 18:**
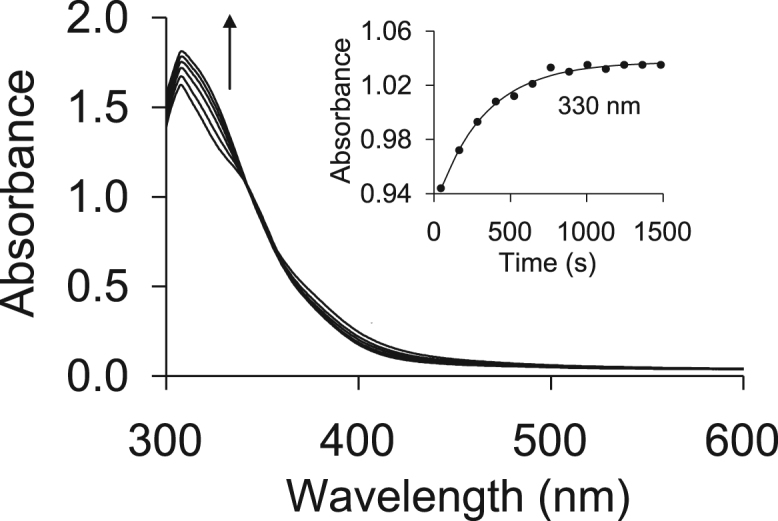
Time-based Uv/vis spectra for the first step in the oxidative addition of CH_3_I onto the Rh(I) metal centre, using the time trace of Rh(H_3_CCOCHCOCH_3_)CO(C_6_H_5_OPPh_2_) (**4**) as an example. The insert shows the absorbance vs time graph measured at 330 nm.

**Fig. 19:**
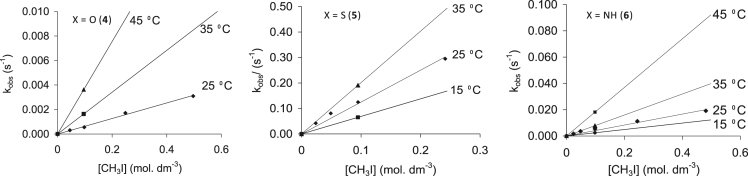
The temperature and methyl iodide concentration dependence of the oxidative addition reaction between CH_3_I and Rh(H_3_CCOCHCOCH_3_)CO(C_6_H_5_XPPh_2_), where X = O (**4**), S (**5**) and NH (**6**) as monitored by UV/vis.

**Fig. 20:**
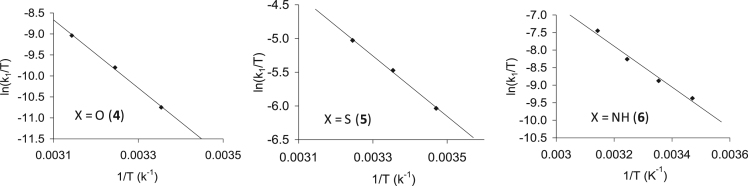
Eyring plots of ln(k_1_/T) vs 1/T for Rh(H_3_CCOCHCOCH_3_)CO(C_6_H_5_XPPh_2_), X = O (**4**), S (**5**) and NH (**6**) measured at temperatures ranging from 15 to 45 °C.

Time-based UV/vis spectra for the oxidative addition of CH_3_I onto the Rh(I) metal centre are shown in Fig. 18, while the temperature dependence and Eyring plots are given in Fig. 19 and Fig. 20, respectively, with the kinetic data obtained from the plots summarised in Table 4.

**Table 4:** Kinetic rate constants, activation parameters and Pauling electronegativity (χ_R_) for the UV/vis-monitored reaction between CH_3_I and **4**, **5** and **6**.

No.	**χ**_**X**_	Temperature (°C)	k_1_ (dm^3^ mol^−1^ s^−1^)	ΔH* (J mol^−1^)	ΔS* (J mol^−1^ K^−1^)	ΔG* (J mol^−1^)^a^
**4**	3.44	25	0.0064	67 (3)	−60 (9)	17.9
		35	0.0171			
		45	0.0378			
**5**	2.58	15	0.6892	37 (2)	−118 (6)	35.2
		25	1.2517			
		35	2.018			
**6**	3.04	15	0.0245	48 (5)	−108 (15)	32.2
		25	0.0417			
		35	0.0798			
		45	0.1850			

a at 25 °C

**Fig. 21:**
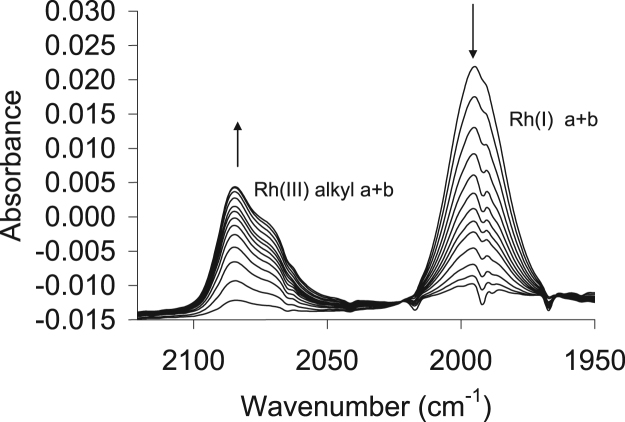
Oxidative addition of CH_3_I to the rhodium complex, Rh(H_3_CCOCHCOCH_3_)CO(C_6_H_5_OPPh_2_), **4**, (shown as an example) monitored by infrared in chloroform at 25 °C. The change in vibration height (at 1995 and 2085 cm^−1^) vs time was used to determine k_obs_.

**Fig. 22:**
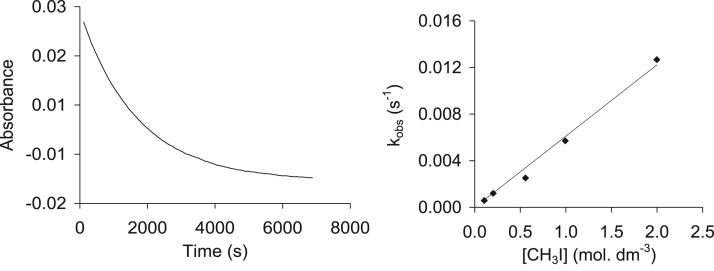
Left: the absorbance vs time graph measuring the decrease in vibration height (2085 cm^−1^) vs time was used to determine k_obs_. Right: the methyl iodide concentration dependence of the oxidative addition reaction between CH_3_I and Rh(H_3_CCOCHCOCH_3_)CO(C_6_H_5_OPPh_2_), (**4**), as monitored by FTIR.

The oxidative addition reaction was also followed by FTIR; Fig. 21 represents the time-based FTIR, while Fig. 22 depicts the absorbance/time graph and concentration dependence graph, as monitored by FTIR. The data obtained the kinetic measurement from the FTIR are given in Table 5, while Table 6 gives a comparative summary of the kinetic data as measured by UV–vis and FTIR.

**Table 5:** Kinetic rate constants and Pauling electronegativity (χ_X_) for the FTIR-monitored reaction between CH_3_I and **4**, **5** and **6**.

No.	X	**χ**_**X**_	k_1_ (dm^3^ mol^−1^ s^−1^)
**4**	O	3.44	0.0061
**5**	S	2.58	1.22
**6**	NH	3.04	0.0444

**Table 6:** The kinetic ate constants of the oxidative addition of methyl iodide onto Rh(H_3_CCOCHCOCH_3_)CO(C_6_H_5_XPPh_2_), where X = O (**4**), S (**5**) and NH (**6**) as obtained by UV/vis and FTIR spectroscopy.

No.	Method	k_1_ (dm^3^ mol^−1^ s^−1^)
**4**	UV/vis	0.0064
	FTIR	0.0061
**5**	UV/vis	1.2517
	FTIR	–
**6**	UV/vis	0.0417
	FTIR	0.0444

## 2 Experimental design, materials and methods

### 2.1 Synthesis

The heteroatomic organophosphorus ligands of the type C_6_H_5_XPPh_2_, where X = O (**1**), S (**2**) and NH (**3**), were prepared according to published procedures [1].

### 2.2 Electrochemistry

Electrochemical measurements were conducted on ca. 0.2 mmol dm^−3^ solutions of the four rhodium(I) complexes in acetonitrile, containing 0.10 mmol dm^−3^ tetra-*n-*butylammonium hexafluorophosphate (Fluka, electrochemical grade) as supporting electrolyte, under a blanket of purified argon, at 25 °C, utilizing a Princeton Applied Research PARSTAT 2273 voltammograph, running PowerSuite (Version 2.58). A three-electrode cell, utilizing a Pt auxiliary electrode, a glassy carbon working electrode, and an Ag reference electrode, was employed. Temperature was kept constant within 0.5 °C. All electrode peak potentials were reported, using the potential of the ferrocene/ferricinium redox couple [FcH/FcH^+^] (*E*^°`^ = 0.00 V) as an internal reference [2]. Successive experiments under the same experimental conditions showed that all formal reduction and oxidation peak potentials were reproducible within 5 mV.

### 2.3 Kinetic measurements

The methyl iodide oxidative addition onto the various rhodium complexes was studied by means of FTIR, at 25 °C in a KCl liquid cell connected to a water bath for temperature control, while monitoring the disappearance of the Rh(I) and formation of the Rh(III) carbonyl peaks. This reaction was also followed using the UV–vis of the dilute rhodium complexes in quartz cuvettes on the Shimadzu UV/Vis spectrometer. At least four temperatures ranges between 15 and 45 °C was monitored, from which the activation parameters ∆H^*^ and ∆S^*^ were obtained. Chloroform was used as solvent and passed through basic alumina just before use to make it acid free. All kinetic measurements were monitored under pseudo first-order conditions with a 500–2000 times molar excess of CH_3_I over the concentration of the rhodium complex. Pseudo first-order rate constants, k_1_, were calculated using MicroMath Scientist 2.0 program.
